# In Vivo Hippocampal Measurement and Memory: A Comparison of Manual Tracing and Automated Segmentation in a Large Community-Based Sample

**DOI:** 10.1371/journal.pone.0005265

**Published:** 2009-04-16

**Authors:** Nicolas Cherbuin, Kaarin J. Anstey, Chantal Réglade-Meslin, Perminder S. Sachdev

**Affiliations:** 1 Centre for Mental Health Research, Australian National University, Canberra, Australian Capital Territory, Australia; 2 School of Psychiatry, University of New South Wales, Sydney, New South Wales, Australia; 3 Neuropsychiatric Institute, Prince of Wales Hospital, Sydney, New South Wales, Australia; University of Regensburg, Germany

## Abstract

While manual tracing is the method of choice in measuring hippocampal volume, its time intensive nature and proneness to human error make automated methods attractive, especially when applied to large samples. Few studies have systematically compared the performance of the two techniques. In this study, we measured hippocampal volumes in a large (N = 403) population-based sample of individuals aged 44–48 years using manual tracing by a trained researcher and automated procedure using Freesurfer (http://surfer.nmr.mgh.harvard.edu) imaging suite. Results showed that absolute hippocampal volumes assessed with these methods were significantly different, with automated measures using the Freesurfer software suite being significantly larger, by 23% for the left and 29% for the right hippocampus. The correlation between the two methods varied from 0.61 to 0.80, with lower correlations for hippocampi with visible abnormalities. Inspection of 2D and 3D models suggested that this difference was largely due to greater inclusion of boundary voxels by the automated method and variations in subiculum/entorhinal segmentation. The correlation between left and right hippocampal volumes was very similar by the two methods. The relationship of hippocampal volumes to selected sociodemographic and cognitive variables was not affected by the measurement method, with each measure showing an association with memory performance and suggesting that both were equally valid for this purpose. This study supports the use of automated measures, based on Freesurfer in this instance, as being sufficiently reliable and valid particularly in the context of larger sample sizes when the research question does not rely on ‘true’ hippocampal volumes.

## Introduction

Imaging studies investigating the human hippocampus have traditionally used manual tracing to measure this structure, which has become the method of choice in most cases. Tracing permits the accurate delineation of the boundaries of the hippocampus, which needs expert training and reference to anatomical landmarks [see 1 for a discussion]. Unfortunately, manual tracing is tedious, resource intensive and prone to human error. These limitations become particularly relevant when large MRI data sets from population based studies must be analysed, arguing for a need for automated measurement.

In addition, the variability in the protocols used to trace the hippocampus has been highlighted in a recent review [2] and therefore the comparability of results across studies can be questioned. For instance, some protocols differ in their inclusion of the subiculum which may increase hippocampal volume by as much as 15% while the brain orientation during tracing can also significantly affect measurements [1].

The ready availability of powerful computer systems and the development of advanced software packages relying on multivariate algorithms and atlases to automatically segment the hippocampus and other structures have progressively ushered these techniques into main stream research. These automated measures have been compared to manual tracings as the reference standard in only a few studies with relatively small sample sizes. Van de Pol et al. [3] compared manually traced to automatically segmented hippocampi (using fluid registration) in 18 participants with Mild Cognitive Impairment assessed at two time points two years apart. They found that the mean atrophy rates did not differ between the two methods, but the intra-class correlation coefficients (ICC) for the longitudinal measure were substantially lower for the manual method (left 0.798, right 0.850) compared to the automated method (left 0.985, right 0.988). They concluded that the fluid registration method they used was more reliable in assessing hippocampal atrophy rates. Another study compared manual tracing and automated segmentation using FreeSurfer (surfer.nmr.mgh.harvard.edu) and IBASPM (thomaskoenig.ch/Lester/ibaspm.htm) of the hippocampus in 21 patients with chronic major depressive disorder and 20 controls [4]. They reported that all methods identified the left hippocampus as significantly smaller in the patient than in the control group and that while the automated measures were significantly larger than the manual measures, the ICC between the manual and automated methods were high (FreeSurfer, left 0.846, right 0.848; IBASPM, left 0.645, right 0.717). A third study [5] designed to validate FreeSurfer against manual tracing reported similar finding for a sample of 134 participants with an ICC between methods of 0.8. Finally, Carmichael et al. [6] compared a number of automated algorithms (comparable to that used in FreeSurfer) to manual tracing of the hippocampus and found that the overlap between measures conducted by two human tracers or between a manual tracer and automated methods was not significantly different, but these analyses were conducted in only six subjects.

Overall, these studies suggest that although differences exist between manual and automated methods, a similar portion of the variance in hippocampal volume appears to be captured by these measures. In addition, due to their high replicability over time, automated methods might have an advantage over manual tracing in longitudinal analyses. Unfortunately, most of the studies reported above were based on relatively small sample sizes and typically did not investigate how the segmented volume differed qualitatively between methodologies. It is also unclear how well automated measures can capture subtle variance in brain volumes (e.g. hippocampus) which could explain variability in other measures sampled from non-clinical cohorts (e.g. memory, cognition) because available studies comparing these manual and automated method have generally investigated absolute differences in volume rather than associations with other non-morphological variables.

The involvement of the hippocampus in memory function has been clearly established. The CA3 hippocampal subfield has been shown to be particularly involved in working and short-term memory [7] while CA1 and CA3 subfields contribute to long-term memory [8]. The subiculum which connects the hippocampus to parahippocampal regions has also been shown to be instrumental in learning and memory [9]–[12]. The exact role of the hippocampus in short- and long-term memory function continues to be hotly debated but it is clear that hippocampal lesions can lead to anterograde [13] and retrograde amnesia [14]. Although there are diverging views as to whether some memory traces are stored in the hippocampus there is widespread agreement that the hippocampus is required for the formation, consolidation and/or retrieval of semantic, autobiographical, episodic, implicit and procedural memories [see 15 for a discussion], [16]. Functional magnetic resonance imaging (fMRI) studies have shown that the hippocampus is involved in encoding and retrieval of memory traces [17], [18] and structural MRI studies have demonstrated that hippocampal volumes in pre-term babies correlates with later working memory deficits [19], increased anterior hippocampal grey matter in postmenstrual women has been found to be associated with improved verbal declarative memory [20], larger posterior hippocampal volumes have been reported in a small sample of London taxi drivers who have a particular need to access stored spatial representation of the environment [21], and in Cushing's syndrome increased hippocampal volume following treatment was associated with improved performance in a word list learning task [22]. In normal ageing and dementia Petersen et al. [23] found significant associations between hippocampal volume and a number of memory measures but no such relationships were detected when the analyses were restricted to cognitively normal individuals. Despite the profuse amount of evidence linking hippocampal structure and function to memory in generally small selected clinical and non-clinical groups we are not aware of any large study relating hippocampal volume to memory performance in a middle-aged non-clinical sample more representative of the population at large.

The aims of this study were to compare volumetric estimates of the hippocampus produced by manual tracing and automatic segmentation using the Freesurfer suite in a large sample of middle-aged participants recruited from a randomly sampled community-based cohort, and to identify potential causes for differences. Additional aims were to determine whether the variances in these measures were comparable and whether they had similar associations with relevant functional measures with a particular focus on working and short-term memory. It was hypothesised that larger hippocampal volumes would be associated with improved working and short-term memory performance. The Freesurfer package was chosen because it is actively developed, has acquired a very good reputation in a relatively short time, is already being used in publications reporting clinical findings, and is freely available and well supported.

## Methods

### Participants

The sample was drawn from the PATH Through Life Project designed to study the risk and protective factors for normal ageing, dementia and other neuropsychiatric disorders [24]. This PATH Project cohort comprised 2530 individuals aged 44–48 years who were residents of the city of Canberra and the adjacent town of Queanbeyan, Australia, and were recruited randomly through the electoral roll. Enrolment to vote is compulsory for Australian citizens. A randomly selected subsample of 656 participants was offered an MRI scan, which 503 accepted, and 431 (85.7%) eventually completed. There were no differences in age, sex, and years of education between those who had an MRI scan and those who did not (p>0.05). One scan was lost due to a technical fault, giving a total number of 430 scans. The reasons for not undergoing an MRI scan after having initially agreed included subsequent withdrawal of consent, medical conditions contradicting MRI, and claustrophobia or other anxiety about the procedure. Age, sex and years of education were recorded during the interview, among other variables. The study was approved by the ethics committees of the Australian National University, Canberra and the University of New South Wales, Sydney, Australia. All participants gave written informed consent to be included in this study.

### MRI acquisition

MRI data were acquired on a 1.5 Tesla Gyroscan scanner (ACS-NT, Philips Medical Systems, Best, The Netherlands). T1-weighted 3-D structural MRI images were acquired in coronal plane using Fast Field Echo (FFE) sequence. About mid-way through this study, for reasons outside the researchers' control, the original scanner (scanner A) was replaced with a similar Philips scanner (scanner B). The scanning parameters were kept essentially the same. The first 164 subjects were scanned on scanner A with TR = 8.84 ms, TE = 3.55 ms, a flip angle of 8°, matrix size = 256×256, slices 160, and the field of view (FOV) 256×256 mm. Slices were contiguous with slice thickness of 1.5 mm. For the remaining 268 subjects scanned on scanner B, the TR = 8.93 ms, TE = 3.57 ms values were slightly different in order to improve image quality, but all other parameters were exactly the same. To ensure the reliability and compatibility of the data, we compared the subjects scanned on the two scanners on sociodemographic and imaging parameters. There were no differences on age (p = 0.377), or years of education (p = 0.588), but more women were inadvertently scanned on scanner B than A (p = 0.003). The volumetric measures of total intracranial volume (TIV), gray matter (GM) volume, white matter (WM) volume, or cerebrospinal fluid (CSF) volume obtained from two scanners did not differ significantly.

### Image analysis

#### Automated segmentation

**Figure 1:**
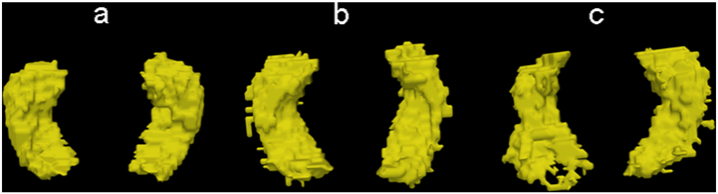
Examplars of automatically segmented hippocampi rated as a) no or minor defects (defects estimated to be smaller than ∼0.5% of total volume) b) moderate defects (∼0.5–5% of total volume), major defects (greater than 5% of total volume).

Volumetric segmentation was performed with the Freesurfer image analysis suite, which is documented and freely available for download online (http://surfer.nmr.mgh.harvard.edu/). This processing includes motion correction, removal of non-brain tissue using a hybrid watershed/surface deformation procedure [25], automated Talairach transformation, and segmentation of the subcortical white matter and deep gray matter volumetric structures (including hippocampus, amygdala, caudate, putamen, ventricles)[5], [26]. The scans of twenty-seven participants were excluded from the sample due to poor scan quality, low signal-to-noise ratio, or movement artefacts which did not allow for normal processing with the standard Freesurfer pipeline. Each segmented volume was inspected by producing a 3D model using the Slicer software package (www.slicer.org) and its quality rated: a) no or minor defects (estimated to be smaller than ∼0.5% of total volume) b) moderate defects (∼0.5–5% of total volume), major defects (greater than 5% of total volume). Exemplars are given in Figure 1.

#### Manual tracing

The volumes of brain anatomical regions were determined by manually outlining the periphery of the ROI on the coronal T1-weighted slices using Analyze 5.0 (Brain Imaging Resource, Mayo Clinic, Rochester, MI, USA). The outlining of the hippocampus always proceeded from anterior to posterior, and was traced according to the protocol described by Watson and colleagues [27]. In addition, the hippocampal tail was manually traced according to the protocol described in detail in Maller et al. [1]. The scans of ten individuals were re-traced to compute an intra-class correlation (ICC) measure. Intra-class correlations were 0.990 for the left and 0.997 for the right hippocampus. While an inter-class correlation measure was not computed for this sample. Such a measure was computed for another sample (using the same operator) which is part of the same larger study and demonstrated very high inter-rater reliability [1].

### Cognitive measures

A number of cognitive measures were used. Episodic memory was assessed based on Immediate and delayed recall of the first trial of the California Verbal Learning Test [28]; verbal working memory was assessed with the Digits Span Backwards task from the Weschler Memory Scale; general cognitive capacity was assessed with the Spot-the-word task, a lexical decision task thought to be an index of premorbid intelligence [29]; information processing speed and attention were assessed with the Symbol-Digit Modalities Test [SDMT; 30].

### Statistical analysis

All statistical analyses were conducted using SPSS 15 (Chicago: SPSS inc.). Volumetric differences between manual and automated measures were evaluated using paired t-tests. Associations between hippocampal volumes obtained by manual or automated techniques and relevant cognitive measures were assessed by multiple regression analyses, entering all variables simultaneously.

## Results

**Figure 2:**
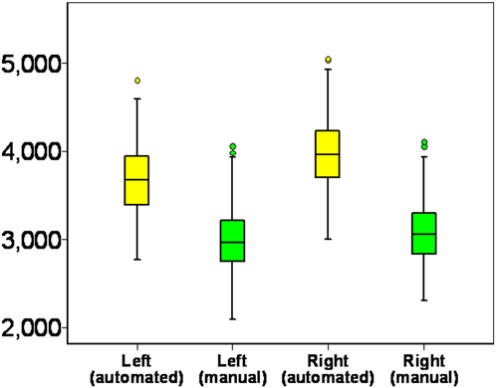
Left and right hippocampal volumes measured by manual tracing (green) and automatic segmentation (yellow). ^**^ significant difference at p<.01.

**Figure 3:**
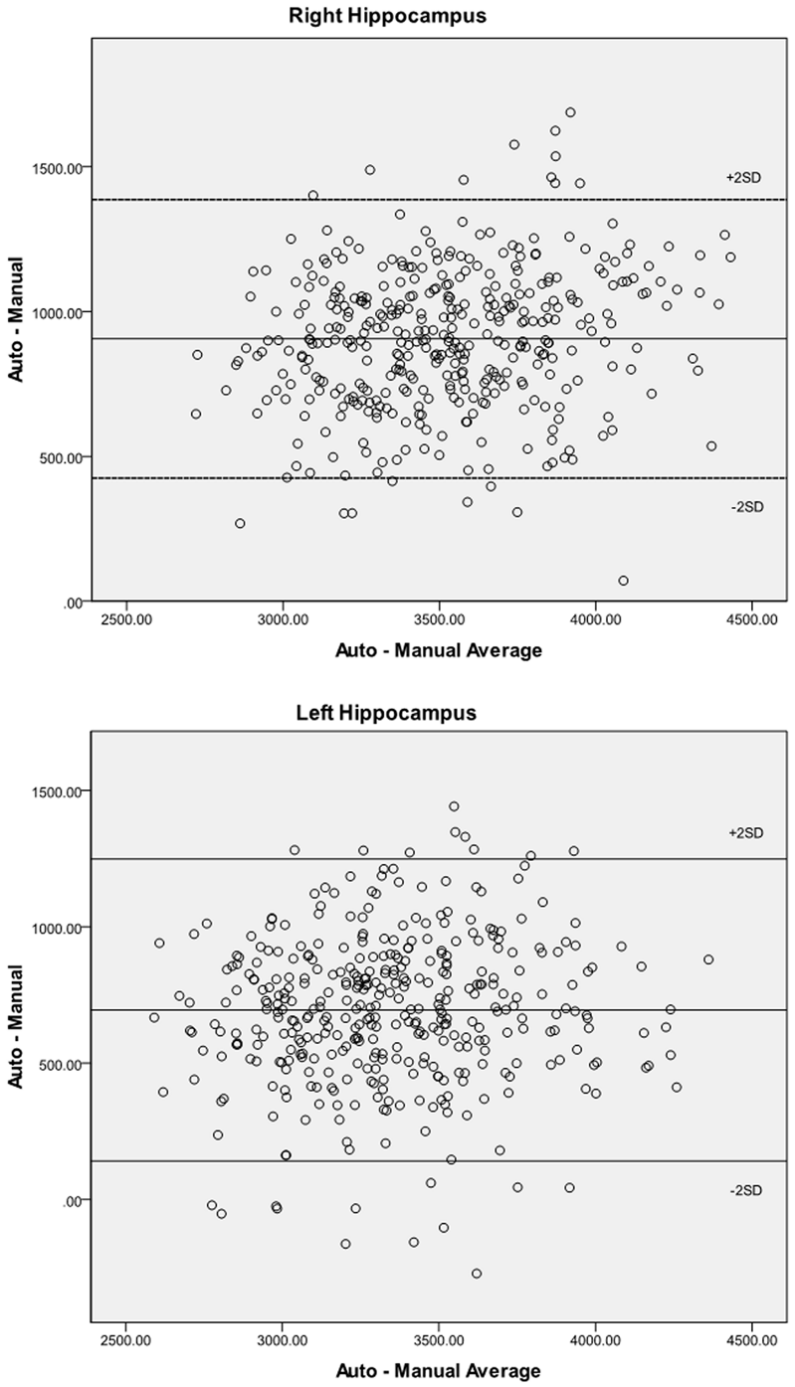
Bland-Altman diagrams plotting the difference in hipocampal volumes computed with the manual and automated methods against their average for each hippocampal pairs for the right (top) and left (bottom) hippocampus. The even scatter suggests there is no systematic error between methodologies beside the difference in absolute volume.

The sample studied was composed of 178 men and 225 women with a mean age of 46.7 years and a mean education level of 14.9 years. Average manually traced volumes were 2992 mm^3^ (SD 355) for the left and 3068 mm^3^ (SD 340) for the right hippocampus. Average automatically segmented volumes were 3688 mm^3^ (SD 372) for the left and 3974 mm^3^ (SD 381) for the right hippocampus (Figure 2). The difference between traced and automatically segmented volumes was significant for both left (t(402) = 27.12, p<.001) and right (t(402) = 35.66, p<.001) hippocampus. Bland-Altman diagrams (Figure 3) plotting the difference in hippocampal volume between methods against their average for each hippocampal pairs show that apart from the absolute difference in volume between the two measurement methods there is no evidence of other systematic error and while some outliers are present, they are few.

Correlations between left and right hippocampi were r = .82 (p<.001) for manual tracing and r = .83 (p<.001) for automatic segmentation.

**Figure 4:**
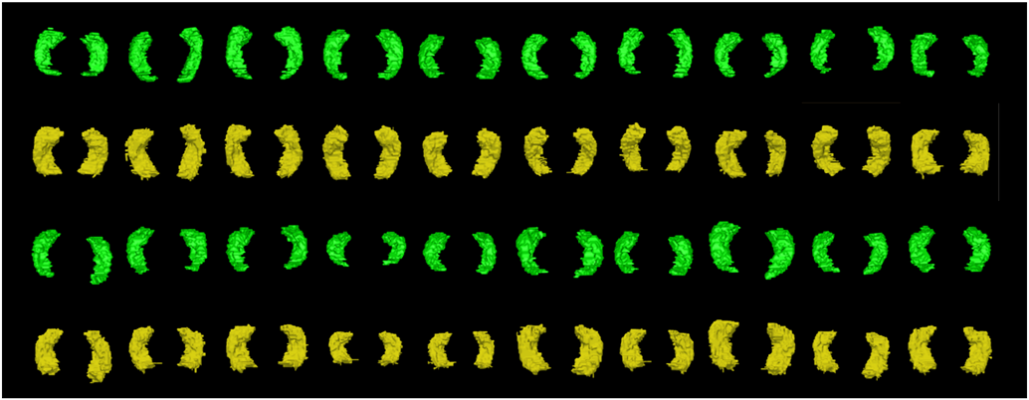
Randomly selected 3D models of manually traced (green) and automatically segmented (yellow) hippocampus pairs. The models were produced using the ITK-Snap software package (www.itksnap.org) for the manual tracings and the Slicer package (www.slicer.org) for the automated measures.

The quality of the automated segmentation was visually assessed and ratings of the 3D hippocampi revealed that 162 pairs had no obvious defects, 182 pairs had minor defects, 46 pairs had moderate defects, and 13 pairs had major defects (see Figure 1). Hippocampi with major defects were excluded from further analyses as they were few, could easily be identified, and would necessitate manual editing in order to be analysed, which was not the focus of the present study. Figure 4 shows a random sample of 3D models of hippocampus pairs based on manual tracings and automated segmentation.

To further investigate the association between manual and automated measures, correlations between measurement methods were computed for the left and right hippocampi and for the different segmentation quality groups, and are presented in Table 1.

**Table 1:** Correlations between hippocampal volumes measured by manual tracing and automated segmentation for different levels of segmentation quality.

Manual Tracing	Automated Segmentation							
	Whole Sample without major defects (n = 390)		No Visible Defects (n = 162)		Minor Defects (<∼0.5% of volume; n = 182)		Moderate Defects (∼0.5%–5% of volume; n = 46)	
	Left	Right	Left	Right	Left	Right	Left	Right
Left	0.714		0.736		0.727		0.606	
Right		0.784		0.784		0.798		0.724

Multiple regression analyses were conducted with manual and automated left and right hippocampal volumes as predicted variables, cognitive variables (immediate and delayed recall, digits backwards, spot-the-word, and SDMT) as predictors, and age, sex, education, and intra-cranial volume as covariates to assess whether the variance of theoretically relevant variables could be explained in a similar pattern by the two measurement methods. The analyses were applied to the whole sample (N = 390) excluding the poor quality scans and to a subsample comprising the scans with no, or minor defects (N = 344). The results of these analyses are presented in Table 2 and show a very good agreement between patterns of association between predictor and predicted variables for both measurement methods. Of particular note, performance on the digit backward task (working memory) was significantly and positively associated with left and right hippocampal volumes measured by manual tracing and with automatic segmentation.

**Table 2:** Multiple regression analyses contrasting association patterns between hippocampal volume (traced or manually segmented) and theoretically relevant individual variables for the whole sample (excluding segmentations with major defects) and for a sub-sample with no or minor segmentation defects.

Predictors		Whole Sample (except major defect; n = 390)				No or Minor Defects (n = 344)			
		Left Hippocampus		Right Hippocampus		Left Hippocampus		Right Hippocampus	
		Manual	Auto	Manual	Auto	Manual	Auto	Manual	Auto
Age	Beta	.046	−.017	.005	−.022	.055	−.017	.005	−.005
	P	.284	.678	.916	.594	.231	.684	.917	.910
Sex	Beta	−.175	−.158	−.065	−.088	−.161	−.166	−.026	−.078
	P	.002^**^	.003^**^	.261	.104	.007^**^	.003^**^	.659	.159
Education	Beta	−.025	−.087	.021	−.106	−.044	−.089	.014	−.108
	P	.600	.050^**^	.659	.019^**^	.372	.052	.784	**.020**
ICV	Beta	.438	.515	.486	.556	.454	.528	.515	.583
	P	.000^**^	.000^**^	.000^**^	.000^**^	.000^**^	.000^**^	.000^**^	.000^**^
Immediate Recall	Beta	.079	−.057	.116	−.017	.086	.003	.115	.035
	P	.326	.448	.154	.823	.310	.967	.179	.655
Delayed Recall	Beta	−.064	.043	−.087	.032	−.068	.035	−.068	.017
	P	.421	.563	.279	.668	.416	.645	.418	.824
Digits Backwards	Beta	.102	.073	.132	.100	.118	.114	.174	.131
	P	.025^*^	**.087**	.004^**^	.022^*^	.016^*^	.011^*^	.000^**^	.004^**^
Spot-The-Word	Beta	−.103	.009	−.077	.014	−.072	.018	−.077	.018
	P	.036^*^	.837	.119	.759	.163	.698	.137	.701
SDMT	Beta	.005	−.027	−.012	−.025	−.029	−.049	−.086	−.066
	P	.915	.532	.794	.561	.557	.283	.085	.150
Adjusted R^2^		.311^**^	.393^**^	.290^**^	.378^**^	.318^**^	.420^**^	.303^**^	.408^**^

Level of significance: ^*^<0.05 ^**^<0.01.

Significant associations are shown in bold.

Due to the presence of significant sex effects in the previous analyses and because gender-specific variation in memory performance have been described in the literature the same regression analyses were conducted in sub-samples stratified by sex. These analyses (Table 3) revealed that in these smaller samples affording less statistical power an association between performance in the digits backward task and hippocampal volume was only detectable in males and for the right hippocampus for both methodological methods. Other notable results were also found. A significant association between education and right hippocampus in males and between education and left hippocampus in females was present but only for the automated measure. Immediate and delayed recall performance was associated with right hippocampal volume in females while the Spot-the-word measure was associated with left hippocampal volume in males but only for the manual measure.

**Table 3:** Multiple regression analyses investigating the relationship between cognitive variables and hippocampal volumes (manual and automated) in samples stratified by sex.

Predictors		Males				Females			
		Left Hippocampus		Right Hippocampus		Left Hippocampus		Right Hippocampus	
		Manual	Auto	Manual	Auto	Manual	Auto	Manual	Auto
Age	Beta	.032	−.015	.011	.016	.060	−.022	−.016	−.027
	P	.650	.828	.869	.823	.365	.731	.805	.656
Education	Beta	.056	−.042	.014	**−.136**	−.111	**−.145**	.023	−.101
	P	.469	.584	.857	**.076**	.124	**.037**	.749	.131
ICV	Beta	**.425**	**.451**	**.457**	**.424**	**.301**	**.441**	**.311**	**.507**
	P	**.000**	**.000**	**.000**	**.000**	**.000**	**.000**	**.000**	**.000**
Immediate Recall	Beta	−.025	−.198	−.080	−.127	.199	.060	**.331**	.090
	P	.844	.106	.513	.300	.107	.615	**.007**	.429
Delayed Recall	Beta	−.012	.137	.081	.146	−.128	−.045	**−.274**	−.079
	P	.923	.261	.505	.233	.298	.702	**.024**	.489
Digits Backwards	Beta	.103	.120	**.170**	**.205**	.113	.052	.110	.037
	P	.185	.114	**.025**	**.008**	.100	.430	.104	.557
Spot-The-Word	Beta	**−.145**	−.030	−.097	−.021	−.081	.053	−.061	.045
	P	**.077**	.702	.219	.796	.272	.453	.405	.509
SDMT	Beta	−.036	.033	−.083	−.055	.065	−.079	.061	−.015
	P	.639	.664	.270	.469	.355	.243	.377	.814
Adjusted R^2^		.172^**^	.210^**^	.219^**^	.205^**^	.105^**^	.171^**^	.134^**^	.237^**^

Level of significance: ^*^<0.05 ^**^<0.01.

## Discussion

The present study sought to contrast measurements of hippocampal volume using manual tracing and automated segmentation using a freely available and widely used software suite, Freesurfer, and to investigate a hypothesised association between hippocampal volume and memory performance. The present findings demonstrate three main points: a) automated hippocampal volumes using Freesurfer are significantly larger than manually traced volumes by an experienced operator b) the variance measured using manual tracing and automated segmentation in Freesurfer appears to have very similar characteristics despite the volumetric differences c) hippocampal volume in mid-life is associated with working memory, particularly in males.

**Figure 5:**
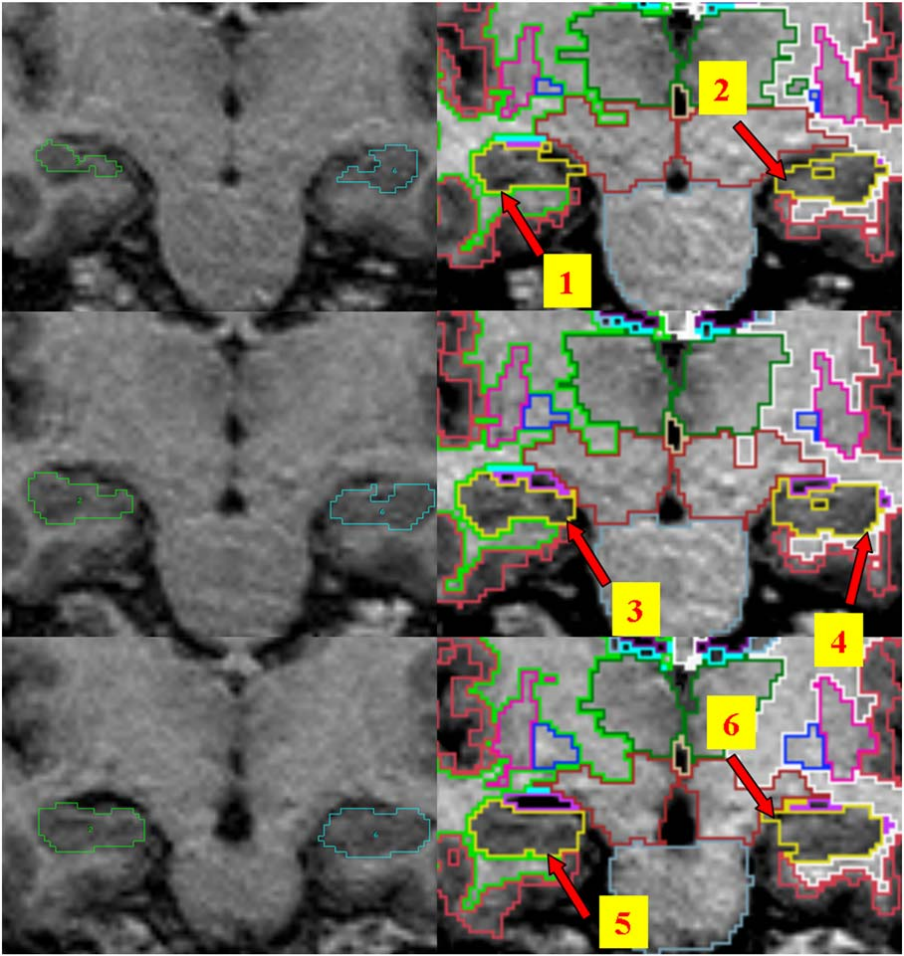
Manual tracing (left) and automated segmentation (right) of the hippocampus showing boundary differences (1 & 6: CSF inclusions; 2 greater subicular/entorhinal inclusion; 4 & 5 greater partial volume inclusion).

Hippocampal volumes measured using Freesurfer were on average 26% larger than those computed using manual tracings. These findings are consistent with those of Tae et al. [4]. Comparisons of the segmentation protocols used for manual tracing and to create the atlas used in Freesurfer did not identify important differences that could account for the present results. Inspection of 2D and 3D models based on both methodologies suggests that these differences are probably due to two main factors. Firstly, the volumes produced using Freesurfer appear uniformly larger (see Figure 4). A close inspection of single slices (see Figure 5) points to a number of causes including an over-inclusion of boundary voxels composed of both grey and white matter (or cerebrospinal fluid) and misidentification of small pockets of CSF as hippocampal tissue. Secondly, although the manual and automated methodologies both include the subiculum, they differ in their assessment of the subicular/entorhinal and parahippocampal boundary for which there is no absolute cut-off (see Figure 4). As a consequence hippocampal volumes produced in Freesurfer include a substantially greater portion of the subiculum/entorhinal/parahippocampal region than the traced volumes. Since the subiculum may account for as much as 15% of hippocampal volume [2], variations in boundaries may account for a large portion of the difference identified between the manual and automated methods in this study. In itself and given the importance of the subiculum/entorhinal/parahippocampal areas in various cognitive processes including memory, differences in inclusion of these structures is not necessarily a negative factor, provided it is done consistently, but it needs to be taken into consideration in the interpretation of results based on automated segmentation with Freesurfer.

Another potential explanation for this difference is that during manual tracing, when confronted with a number of voxels sharing volume between tissue classes operators may decide to alternatively include and exclude such voxels to produce neither over- nor under-inclusion. Such an approach is computationally more difficult in an automated computer package and rules are probably more likely in such cases to lead to either over- or under-inclusion.

It should also be noted that, as mentioned above, although no substantial differences could be identified between the segmentation protocol used for manual tracing and that used to create the template on which the Freesurfer segmentation algorithm is based, the latter segmentation protocol only partly determines the quality of the template with scan number and diversity in individual characteristics (age, education, health, etc.) being other major determinants. Consequently, it may be that computing a template based on a greater number and variety of scans could solve the present discrepancy in volume. Alternatively, the use of varying templates for different age-groups, perhaps one per decade, might address differing segmentation challenges across the lifespan.

While these volume differences are not optimal, they are not necessarily in themselves a major problem unless the research question is interested in absolute volume rather than relationships between volume and other variables of interest (e.g. cognitive performance on a specific task). However, it does mean that the use of different measurement methodologies across waves of measurement in a longitudinal study will not produce accurate measures of structural shrinkage, although the variance in atrophy may not be affected.

Apart from absolute volume, and possibly of greater importance in many studies, the variance associated with manual tracing and automated segmentation was compared in the present sample in three different ways. First, correlations between the two measures were computed and showed good correspondence especially given the greater inclusion of the subiculum/parahippocampal region in the automated method with correlations of between 0.7 and 0.8 and the variability of the two measures was very similar (Figure 2). Bland-Altman diagrams (Figure 3) also show that no systematic error is present between measurement methods apart from the difference in absolute volume. Secondly, if the two methodologies are equally reliable (i.e. inter-rater reliability), it would be expected that the association between left and right hippocampi would be similar despite the different inclusion of certain structures (subiculum) and boundary voxels. This was indeed the case with correlation between left and right hippocampi of 0.82 for manual tracing and 0.83 for the automated measure.

The third way in which congruence in variance between measures was assessed is by comparing the pattern of explained variance in hippocampal volume by a number of theoretically relevant predictors including age, sex, education, intra-cranial volume, immediate and delayed recall, a backward counting task, a lexical task (Spot-the-word), and a speed of processing and attentional task (Symbol-Digit Modalities Test). The results of logistic regression analyses (Table 2) show that the association between hippocampal volume and predictors is very similar across the two measurement methods with intra-cranial volume and performance on the digit-backward task being significant predictors of both left and right hippocampal volume for the two measures and sex being a significant predictor for the left hippocampus only but again this was true for both measures. Some differences were also found with the automated measure demonstrating a small but significant association with sex, and the manual measure with the Spot-the-word task, although marginally and only for the left hippocampus. Together, these findings suggest that the automated measure is not less sensitive than the traced measure, at least in the present sample.

Another aim of this study was to investigate a hypothesised association between larger hippocampal volume and working and short-term memory. Multiple regression analyses showed that performance on the digits backward task, which involves recalling strings of numbers of increasing length in reverse order, was significantly associated with hippocampal volume for both measures. In contrast, no such association was found with the immediate or delayed recall tasks which involve recalling a shopping list of sixteen items without delay or following a distracter task with a one minute delay. These findings suggest that the association between item and item position or the storage of more abstract stimuli such as digits compared to words might be particularly sensitive to hippocampal function and structure. Due to reported sex differences in memory function in the literature [31] and the presence of a sex effect in the main analysis, posthoc analyses with stratisfication for sex were conducted. These analyses revealed a significant association between digit backward performance only for the right hippocampus in males. In females a significant association with right hippocampal volume was present for immediate and delayed recall. These findings are particularly interesting because Otero et al. [31] found that women performed better in immediate and delayed object recall as well as in delayed verbal memory while men performed better in digit span which appears consistent with the present results. Gender differences in Hippocampal size and memory function have also been shown to be influenced by the menstrual cycle [20], [31]. However, this influence was not tested in the present study but may contribute to gender differences. In addition lateralised effects in memory function have been previously documented [16], [32] with left hippocampal lesions being more often associated with verbal memory deficits while right hippocampal lesions are more often associated with visuo-spatial deficits. In a detailed review of the episodic/visuo-spatial memory literature Burgess et al. [33] concluded that the left hippocampus is probably specialised for episodic, autobiographical memory while the right hippocampus is more involved in object-location memory. Although the association between right hippocampus and the digits backwards task appears consistent with a right-lateralised object-location memory system, the association between right hippocampus and immediate and delayed recall is not unless a visuo-spatial strategy was used to remember the shopping list. Unfortunately this hypothesis cannot be tested in the present study. An alternate, speculative explanation might be that in these middle-aged individuals right hippocampal function assists or compensates for decline in left hippocampal function. It is recognised that the pathological processes leading to dementia and cognitive decline in ageing start earlier and are more extensive in the left medial temporal lobe [34] and that early signs can already be observed in post-mortem studies in the third or fourth decade of life [35], [36]. Thus it is possible that later in life larger right hippocampal volumes contribute significantly to processes that would be more reliant on the left hippocampus earlier in the lifespan. This hypothesis is partly supported by the findings of a recent study investigating regional grey matter volume and metabolism in 45 healthy subjects aged 20–83 years [37]. Kalpouzos et al. found that the hippocampus was one of the structures least affected by age-related atrophy and decrease in metabolism, but interestingly they found that the left was more affected than the right hippocampus and that the anterior hippocampus which appears to be more involved in episodic memory encoding was spared while the posterior hippocampus with greater involvement in retrieval was more affected [38]. These findings might suggest that the present association between hippocampal volume and memory could be due to a retrieval deficit mediated by greater posterior hippocampal atrophy. This hypothesis cannot be tested in the present study but future longitudinal research should investigate this question.

This study had a number of limitations. It was performed with participants in a narrow age-range which limits the power of our analyses to detect an age-dependent effect and somewhat limits the generalisability of these findings particularly to older cohorts where major differences in brain atrophy can usually be detected. But a narrow age range is also a strength since differences in hippocampal volume are likely to be smaller in such a homogenous cohort and therefore would tend to produce smaller correlations and be more conservative. Another limitation is that this study only investigated one brain structure. The performance of Freesurfer is likely to vary across different regions of interest. However, the hippocampus is one of the more complex subcortical structures to segment and therefore improved reliabilities might be expected for other structures such as the putamen or the ventricles. Another possible limitation is that a small number of scans were excluded from analysis due to major defects during automatic segmentation. If defects were associated with pathology it could diminish the usefulness of automatic segmentation in clinical and diagnostic studies and particularly in those investigating pathologies where hippocampal lesions are expected (e.g. Alzheimer's disease, hippocampal sclerosis). However, inspection of scans with defects does not suggest this to be the case but rather that defects relate to scan quality and artefacts. This study also had several strengths. It was conducted in a large generally healthy community-based sample that is likely to be representative of the general population. Moreover, this study investigated middle-aged participants with full, rounded hippocampi and relatively little atrophy which makes boundaries more difficult to detect and therefore test the limits of the segmentation algorithms. It is possible the agreement between methods would be different in older samples where more atrophy is present. Since clinical and older samples typically present with substantial hippocampal atrophy which is likely to be associated with clearer boundaries and therefore better segmentation, improved reliabilities might be expected in these populations.

The Freesurfer package was chosen due to a number of factors including free and wide availability, active development, good support, and its broader use particularly in clinical research. However, other semi-automated techniques have also shown good performance when compared with manual tracing [39].

In conclusion, the present findings suggest that automated segmentation of the hippocampus using Freesurfer differs in significant ways from manual tracing particularly in that it produces substantially larger volumes. Some but not all this difference can be explained by variation in the amount of the subiculum/entorhinal/parahippocampal regions included while the rest seems to be due to a relatively uniform over-inclusion of boundary voxels and some CSF. Despite these differences, the amount and quality of the variance in these measures appears to be very similar. Consequently, these findings validate Freesurfer segmentation as a measure of hippocampal volume which, at least in large samples with good quality scans, can be recommended as a reliable option. In our view, processing with Freesurfer can also be recommended in smaller samples provided each segmented structure is carefully screened and major defects are manually corrected. However, where scan quality is poor or very variable or when major localised lesions are present (e.g. major infarction) manual tracing may be a preferable option.

The present findings also demonstrate a positive association between hippocampal volume and a measure of working memory in a sample of healthy middle-aged individuals which confirms a relationship between size and function which can be difficult to demonstrate in non-clinical groups.
